# Impact of analgesic techniques on early quality of recovery after prostatectomy: A 3‐arm, randomized trial

**DOI:** 10.1002/ejp.2020

**Published:** 2022-08-21

**Authors:** Christian M. Beilstein, Markus Huber, Marc A. Furrer, Lukas M. Löffel, Patrick Y. Wuethrich, Dominique Engel

**Affiliations:** ^1^ Department of Anaesthesiology and Pain Medicine Inselspital Bern University Hospital University of Bern Bern Switzerland; ^2^ Department of Urology Inselspital Bern University Hospital University of Bern Bern Switzerland

## Abstract

**Background:**

Prostatectomy is associated with relevant acute postoperative pain. Optimal analgesic techniques to minimize pain and enhance recovery are still under investigation. We aimed to compare the effect of three different analgesic techniques on quality of recovery.

**Methods:**

This investigator‐initiated, prospective, randomized, three‐arm, parallel‐group, active‐controlled, interventional superiority trial was performed in a Swiss teaching hospital from 2018 to 2021. Consecutive patients undergoing open or robotic‐assisted radical prostatectomy were randomized to spinal anaesthesia (SSS, bupivacaine 0.5% + fentanyl), bilateral transversus abdominis plane block (TAP, ropivacaine 0.375% + clonidine) or systemic administration of lidocaine (SA, lidocaine 1%) in addition to general anaesthesia. Primary outcome was quality of recovery 15 (QoR‐15) score on postoperative day one compared to baseline. Secondary outcomes were QoR‐15 at discharge, postoperative nausea and vomiting, pain scores, return of gastrointestinal function and use of rescue analgesia.

**Results:**

From 133 patients, 40 received spinal anaesthesia, 45 TAP block and 48 systemic analgesia. QoR‐15 scores did not differ on day 1 (*p* = 0.301) or at discharge (*p* = 0.309) when compared to baseline. QoR‐15 changes were similar in all groups. At discharge, median QoR‐15 scores were considered as good (>122) in all groups: SSS 134 [IQR 128–138]; TAP 129 [IQR 122–136] and SA 128 [IQR 123–136]. There were no significant differences in the other secondary outcomes.

**Conclusions:**

Quality of recovery on postoperative day one compared to baseline did not differ if spinal anaesthesia, TAP block or systemic administration of lidocaine was added to general anaesthesia.

**Significance:**

Optimal analgesic techniques to enhance recovery after prostatectomy are still under investigation. In this 3‐arm randomized controlled trial, addition of spinal anaesthesia or transversus abdominis plane block to general anaesthesia did not improve quality of recovery after radical prostatectomy compared to less invasive intravenous lidocaine infusion (standard of care/control group). Quality of recovery at the time of discharge was considered as good in all three groups.

## 1 INTRODUCTION

Radical prostatectomy (ORP) is associated with relevant acute postoperative pain. Even after less invasive robotic‐assisted laparoscopic radical prostatectomy (RARP), morphine consumption is comparable to ORP (Webster et al., 2005). Poor pain control may delay recovery, result in chronic pain and increase morbidity. Therefore, optimal postoperative pain management is a key factor for enhanced recovery after surgery. In the last years, multimodal analgesia concepts have been developed to optimize patient comfort and achieve fast functional recovery with fewest possible side effects (McEvoy et al., 2017). ORP has early been identified as a major procedure needing effective pain therapy, making it nowadays one of the least painful procedures when comparing 179 surgeries (Gerbershagen et al., 2013).

Nevertheless, Joshi et al. identified insufficient evidence to develop a protocol for optimal pain control in patients undergoing ORP or RARP as most studies only assessed unimodal analgesia but not multimodal concepts (Joshi et al., 2015).

The application of continuous intravenous lidocaine to reduce pain scores after open prostate surgery has first been published almost 25 years ago and is considered standard of care in our institution due to its longstanding excellent results (Groudine et al., 1998).

Besides lidocaine, a plethora of different agents like clonidine, ketamine or gabapentinoids have been studied with conflicting results (Joshi et al., 2015). Most promising was a multimodal approach using acetaminophen, celecoxib and pregabalin for RARP, decreasing intra‐ and postoperative morphine requirements (Trabulsi et al., 2010).

The second approach for multimodal analgesia is the addition of regional anaesthesia to systemic medication. Compared to general anaesthesia, spinal anaesthesia plus sedation reduced length of stay and pain in the postoperative care unit after radical retropubic prostatectomy (Salonia et al., 2004). Intrathecal morphine reduced postoperative pain after ORP (Andrieu et al., 2009; Brown et al., 2004; Nuri Deniz et al., 2013) and after RARP (Bae et al., 2017).

Bilateral transversus abdominis plane (TAP) block is associated with fewer risks than neuroaxial anaesthesia. It lowered pain scores in RARP (Maquoi et al., 2016), but results were conflicting for ORP (Elkassabany et al., 2013).

In summary, there is sufficient evidence of the superiority of multimodal over unimodal analgesia, but a direct comparison of intrathecal analgesia, TAP block or systemic administration of lidocaine has not been performed to date.

In addition, trials published so far mainly focused on pain scores and opioid requirements after surgery, omitting important aspects of recovery such as the return of gastrointestinal function or patient‐reported outcome measures.

Hence, there is a need for more procedure‐specific studies not only comparing pain and analgesic requirements but also assessing quality of recovery. The quality of recovery (QoR) questionnaire is a patient‐centred, global measure of overall health status postoperatively and has been gaining attention in recent years (Myles, 2016b). The short form of the original QoR‐40 is the QoR‐15 questionnaire, consisting of 15 questions with 0 to 10 points each, resulting in a maximum of 150 points. It is well established, validated and fulfils the requirements to be used as an outcome measurement instrument in clinical trials (Kleif et al., 2018; Stark et al., 2013). Its German version has recently been validated (Kahl et al., 2021). Values above 122 points are considered to be good (Kleif, 2018), and the minimal clinically important difference has been reported to be 8 for the QoR‐15 (Myles et al., 2016). This has recently been corrected to 6 (Myles, 2021).

Therefore, the objective of this RCT was to compare the quality of recovery using the QoR‐15 questionnaire in prostatectomy patients treated with three different, multimodal analgesic concepts.

## 2 METHODS

### 2.1 Ethics

The study (KEKBE BASEC N° 2018–00632) was approved by the Ethics Committee of the Canton Bern, Switzerland (Chairperson Professor Ch. Seiler) on 12 July 2018. It was prospectively registered at ClinicalTrials.gov (identifier: NCT03618693; date of registration: 7 August 2018) and conducted in compliance with the Declaration of Helsinki and Good Clinical Practice. All patients provided written informed consent prior to inclusion in the study.

### 2.2 Study design

This was an investigator‐initiated, prospective, randomized, three‐arm, parallel‐group, active‐controlled, interventional superiority trial conducted at the Department of Urology, Bern University Hospital, Switzerland, between 15 August 2018 and 10 December 2021. The reporting complies with the recommendations of the CONSORT statement.

### 2.3 Patients

Consecutive patients who presented for ORP or RARP were screened for eligibility. Participants fulfilling the following inclusion criteria were eligible: >18 years old, eGFR >40 ml min^−1^ and normal liver function. Exclusion criteria were contraindications to the study drugs, regular use of antiemetics, laxatives, analgesics or chronic pain, drug or alcohol abuse, inability to follow the study procedures, psychiatric disorders, refusal or contraindication for regional analgesia.

### 2.4 Interventions

Patients allocated to the spinal analgesia group (SSS) received a single intrathecal injection of bupivacaine 0.5% with 20 μg fentanyl in a sitting position at the lumbar level L3/L4 or lower using a medial approach. Dose of bupivacaine 0.5% applied was 10 mg if older than 70 years or 10 to 15 mg depending on body height if less than 70 years old. Spinal anaesthesia was performed shortly before induction by the senior anaesthesiologist in charge using a 25 gauge pencil point cannula (Pencan®, B Braun Melsungen AG). The effect was assessed prior to induction (feeling of warmth, beginning of paralysis).

Patients allocated to the TAP group received a bilateral block after induction by a senior anaesthesiologist with high expertise, using 20 ml ropivacaine 0.375% combined with 75 μg clonidine. All blocks were performed under ultrasound guidance (Philips Spark®, Philips Health System). The probe was positioned in the mid‐axillary line between the iliac crest and the costal margin, and a 22‐gauge needle (Ultraplex 360®, B Braun Melsungen AG) was inserted in an anterior to posterior direction using an in‐plane technique. The solution was injected on each side in the appropriate plane, separating the transversus abdominis and obliquus internus muscles (Elkassabany et al., 2013; Maquoi et al., 2016). The clinical effect of the TAP block could not be assessed, but local anaesthetic disposition in the correct space was confirmed by two doctors.

Patients allocated to the active control group (i.e., systemic analgesia, SA) received the standard of care for prostatectomy at our institution: concomitant systemic administration of i.v. lidocaine. A bolus of lidocaine (1.5 mg kg ideal body weight [IBW]^−1^) was administered during induction followed by an infusion of 1.5 mg kg IBW^−1^ h^−1^ for 24 h (Daykin, 2017).

After extubation, all patients were transferred to the intermediate care unit (IMC) for at least one night. A nurse (blinded to the randomization) titrated i.v. fentanyl in 25 μg increments until pain scores were below 4 (breakthrough pain treatment).

### 2.5 Standardized perioperative management

All patients were allowed oral intake until midnight before surgery and were encouraged to drink clear fluids until 2 h before induction of anaesthesia. Carbohydrate loading (2 times 400 ml Preload®, Nestlé Health Science) was prescribed for the evening before, respectively, 2 h before arrival to the operating room. No sedative premedication was administered.

Standard monitoring included electrocardiography, nasopharyngeal temperature measurement, pulse oximetry and invasive arterial blood pressure monitoring. Anaesthesia was induced with i.v. propofol (2 mg kg^−1^), fentanyl (2 μg kg^−1^) and rocuronium (0.6 mg kg^−1^) and maintained with desflurane at an age‐corrected minimum alveolar concentration of 0.6. Normothermia was maintained using an air warming system (Bair Hugger®, 3 M‐Switzerland). Intra‐operative maintenance of anaesthesia and analgesia was performed according to our daily practice and internal recommendations with repetitive administration of fentanyl for clinical signs of intra‐operative pain (blood pressure or heart rate increase exceeding 20% when compared to post‐induction baseline). Fluid administration included a maintenance infusion of Ringer's lactate (3 ml·kg BW^−1^·h^−1^) and the substitution of blood loss with Ringer's lactate.

All patients received postoperative multimodal analgesia starting at the end of surgery with ketorolac 30 mg i.v. three times per day for 48 h, metamizol 1 g (i.v./per oral) four times per day and paracetamol 1 g (i.v./per oral) four times per day. Rescue medications were additional boluses of fentanyl i.v. (during surgery and intermediate care unit stay) or oxycodone 5 to 10 mg per oral every 3 to 4 h on the normal ward.

Surgery was performed in a standardized fashion, with the patient in a 30° head‐down position (Burkhard et al., 2006; Kessler et al., 2007). The decision regarding the type of surgical approach (ORP or RARP) was made by the senior urologist in charge and accorded to our internal recommendations and the prostate cancer centre certification. The orogastric tube was removed at the end of the procedure. PONV prophylaxis was performed based on the Apfel's score and included 4 to 8 mg i.v. dexamethasone at induction (score = 1) and additional 4 mg i.v. ondansetron at the suture of the skin (score >1).(Apfel et al., 1999).

PONV was managed first line with i.v. ondansetron 4 mg or in case of persistence i.v. droperidol 0.625 mg every 6 h. The following ERAS program guidelines were implemented: use of chewing gum was encouraged, clear drinks were allowed the same evening after surgery as well as bedside mobilization. Oral fluids and meals included energy drinks on postoperative day (POD) 1. On POD 1, longer mobilization periods including walking and sitting on a chair were encouraged.

### 2.6 Primary and secondary outcomes

The primary objective was to evaluate the impact of three different analgesic concepts: spinal single shot (SSS), TAP block (TAP) or systemic administration of lidocaine (SA, serving as control group) on early postoperative quality of recovery, assessed using the QoR‐15 questionnaire. The primary endpoint was the change in QoR‐15 score from preoperative (day of admission) to POD 1.

QoR‐15 was selected because it is a short, easy‐to‐perform, patient‐reported outcome questionnaire which incorporates five dimensions of patient's health (support, comfort, emotions, physical independence and pain). It is a shortened and validated version of the QoR‐40 questionnaire. It includes 15 items with scores ranging from 0 to 10, resulting in a maximum of 150 points (Kleif et al., 2018; Stark et al., 2013). It has recently been validated in German (Kahl et al., 2021). Values above 122 points are considered to be good (Kleif, 2018), and the minimal clinically important difference has initially been reported to be 8 for the QoR‐15 (Myles et al., 2016). This has recently been corrected to 6 (Myles, 2021). For this trial, an even more conservative approach of a minimal clinically important difference of 10 for the QoR‐15 was used (see determination of sample size).

Secondary objectives were changes in QoR‐15 from preoperative to discharge; incidence of PONV 6, 24 and 48 h postoperatively; pain scores (pain at rest; ‘deep’ visceral pain at rest [considered as pain in the urethra or pelvic pain]; pain during coughing or mobilization) according to the numeric rating scale (NRS) at 6, 24 and 48 h postoperatively; return of gastrointestinal function (first flatus, first defecation, tolerance to food); use of opioids intra‐ and postoperatively, conversed to oral morphine milligram equivalents (MME) for POD 1 and POD 2.

## 3 STATISTICS

### 3.1 Determination of sample size

This trial was a three‐way comparison, and all pairwise comparisons were of interest. The sample size calculation itself was based on the difference between the SSS group and TAP group at POD 1, which was expected to be more difficult to detect than the differences of either to the SA group. Considering a minimal detectable difference in the mean QoR‐15 score of 10 between preoperative and POD 1 and a standard deviation of 14, 43 patients per group were necessary based on a t‐test for independent groups with common variance (Myles, 2016a; Myles et al., 2016). For a significance level of 0.0167 (2‐tailed) accounting for multiple comparisons, we derive a power of 81.2%. Considering a dropout frequency of a bit more than 10%, 48 patients per group were recruited (total of 144 patients). The calculations were made with GPower 3.1 and were based on a t‐test for independent groups with common variance.

### 3.2 Randomization and blinding

Randomization was performed using a computer‐generated list with blocks of 12 patients; allocation was left in concealed opaque and numbered envelopes. Patients were strictly included in ascending order. The list was concealed at the research unit of the Department of Urology. Stratification to ORP or RARP was aimed (1:1). Blinding was only possible for outcome assessors and data analysts.

### 3.3 Summary statistics

Continuous data were examined for normality with the Shapiro–Wilk test and were expressed as mean values and standard deviations when normally distributed or as medians and interquartile ranges (IQR) otherwise. Categorical data were summarized with counts and frequencies and compared using the chi‐square test; a simulated chi‐square test was employed in case of low cell entries.

### 3.4 Data analysis

Data were analysed on a modified intention‐to‐treat basis. In terms of QoR‐15 outcome, the primary endpoint (∆QoR‐15: POD 1—preoperative), secondary endpoints (∆QoR‐15: discharge—preoperative and ∆QoR‐15: discharge—POD 1) were examined using the Kruskal‐Wallis rank sum test. Post‐hoc comparisons between treatment groups were performed only when the global Kruskal‐Wallis rank sum test was statistically significant. Accounting for the repeated measure design of this study, a generalized linear mixed‐effect model (GLMM) was used to examine the treatment * time point interaction for the longitudinal observations of the endpoints pain (NRS score (at rest, deep visceral and during mobilization/coughing), rescue analgesics (binary), fentanyl (binary), PONV (binary) and gastrointestinal function (binary and categorical)) in order to investigate whether the treatment groups differed in their overall time evolution in these endpoints. Given the bounded range of the NRS scale, the NRS variables were transformed into the (0,1) interval, and the GLMM was computed with a beta distribution (Smithson & Verkuilen, 2006). For binary variables, the GLMM was computed with a binomial distribution.

A *p*‐value of <0.5 was considered statistically significant for global statistical tests. Accounting for multiple comparisons, a *p*‐value <0.0167 was considered statistically significant for pairwise post‐hoc analyses. All computations were performed with R version 4.0.2 (The R Core Team [2020], R: A language and environment for statistical computing. R Foundation for Statistical Computing, Vienna, Austria. URL https://www.R‐project.org/).

## 4 RESULTS

**FIGURE 1:**
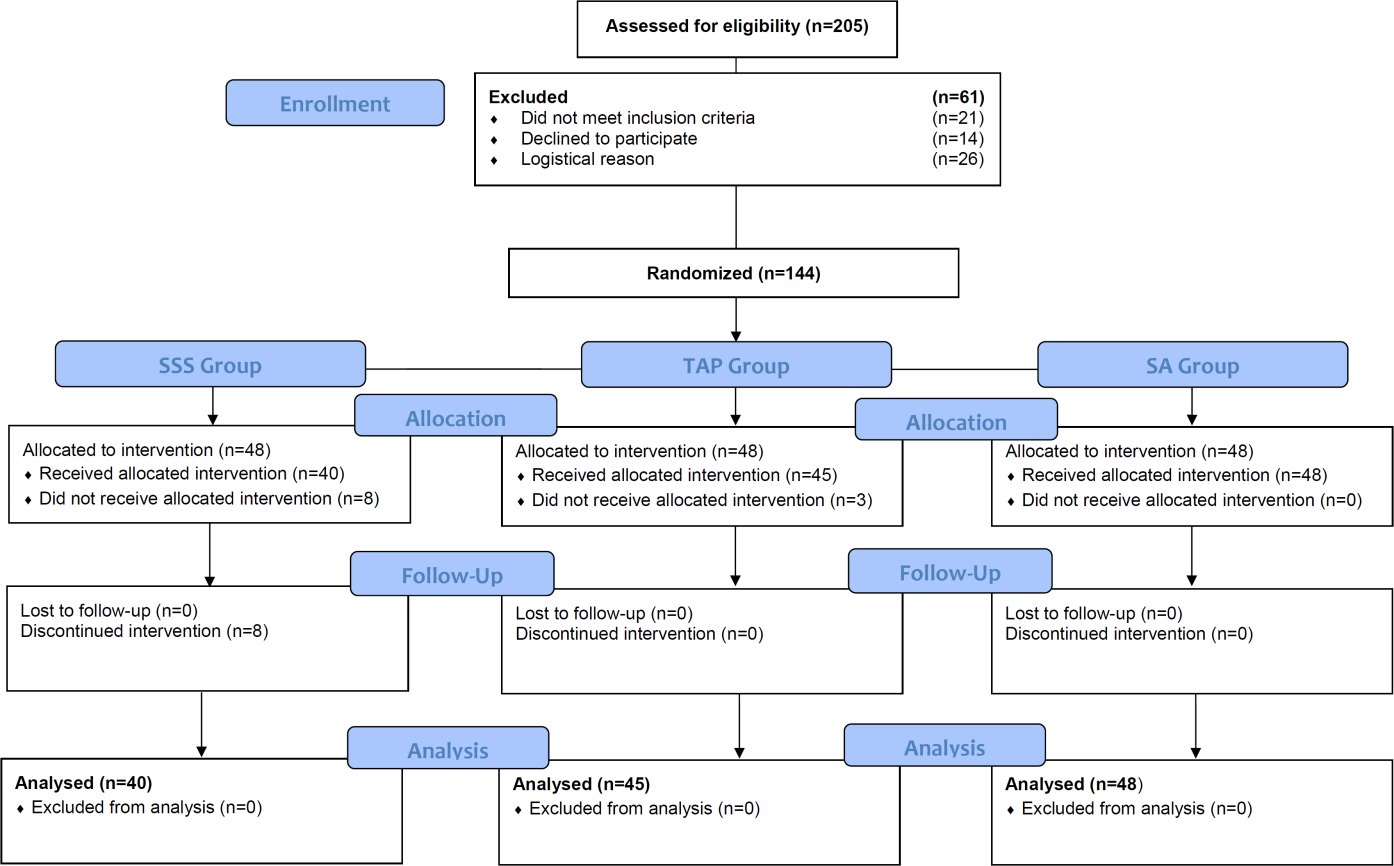
CONSORT flow chart.

Of 205 consecutive patients scheduled for radical prostatectomy, 144 were randomized. Of the patients enrolled, 8 patients in the SSS group (secondary refusal of spinal anaesthesia) and 3 in the TAP group (inability to perform block due to anatomical issues) were excluded (Figure 1). The preoperative baseline characteristics were similar between groups (Table 1). A total of 133 patients were included in the final analysis (SSS, *n* = 40; TAP, *n* = 45; SA, *n* = 48). The median intrathecal bupivacaine dose was 12 mg (10–13). All patients in the TAP group received 150 mg ropivacaine with 150 μg clonidine. The median intraoperative dose of systemic administration of lidocaine was 542 mg (450–607) corresponding to a median dose of 100 mg h^−1^.

**TABLE 1:** Baseline data

	SSS	TAP	SA
	*N* = 40	*N* = 45	*N* = 48
Preoperative data			
Age (years)	67.5 [61.0; 72.0] (*N* = 40)	68.0 [63.0; 72.0] (*N* = 45)	67.5 [64.0; 72.0] (*N* = 48)
Weight (kg)	80.6 (10.3) (*N* = 40)	83.2 (13.0) (*N* = 45)	84.1 (12.8) (*N* = 48)
Height (m)	1.75 [1.72; 1.82] (*N* = 40)	1.75 [1.70; 1.82] (*N* = 45)	1.75 [1.70; 1.80] (*N* = 48)
BMI (kg m^−2^)	24.8 [23.5; 28.2] (*N* = 40)	27.1 [24.0; 28.9] (*N* = 45)	27.8 [24.6; 29.4] (*N* = 48)
ASA			
1	2/39 (5.13%)	1/44 (2.27%)	0/48 (0.00%)
2	30/39 (76.9%)	35/44 (79.5%)	38/48 (79.2%)
3	7/39 (17.9%)	8/44 (18.2%)	10/48 (20.8%)
Ischemic heart disease (Yes)	5/38 (13.2%)	6/45 (13.3%)	5/48 (10.4%)
Hypertension (Yes)	17/40 (42.5%)	29/45 (64.4%)	24/47 (51.1%)
Diabetes mellitus (Yes)	3/40 (7.50%)	4/45 (8.89%)	5/46 (10.9%)
Smoking (Yes)	2/39 (5.13%)	7/45 (15.6%)	4/47 (8.51%)
COPD (Yes)	0/38 (0.00%)	3/45 (6.67%)	1/46 (2.17%)
Asthma (Yes)	0/39 (0.00%)	1/42 (2.38%)	0/47 (0.00%)
Medications			
Betablockers (Yes)	4/40 (10.0%)	4/45 (8.89%)	3/48 (6.25%)
Anti‐hypertensives (Yes)	15/40 (37.5%)	27/44 (61.4%)	21/48 (43.8%)
Statine (Yes)	8/40 (20.0%)	5/45 (11.1%)	9/48 (18.8%)
Aspirin (Yes)	4/39 (10.3%)	4/45 (8.89%)	9/48 (18.8%)
Laboratory tests			
Creatinine (μmol/L)	84.0 [77.5; 93.5] (*N* = 39)	85.0 [77.0; 98.0] (*N* = 45)	83.0 [77.0; 89.2] (*N* = 48)
Hb (g/L)	146 [140; 153] (*N* = 39)	146 [140; 152] (*N* = 45)	144 [138; 150] (*N* = 48)
Hkt (%)	41.8 (3.35) (*N* = 39)	41.6 (2.52) (*N* = 45)	42.0 (2.94) (*N* = 48)
Tc (U/L)	248 [220; 285] (*N* = 38)	225 [193; 240] (*N* = 45)	234 [212; 264] (*N* = 48)
Quick (%)	100 [91.0; 100] (*N* = 38)	98.0 [89.5; 100] (*N* = 43)	98.0 [91.8; 100] (*N* = 48)
Surgical data			
Prostatectomy			
Open	19/40 (47.5%)	23/45 (51.1%)	28/48 (58.3%)
Robotic assisted	21/40 (52.5%)	22/45 (48.9%)	20/48 (41.7%)
Duration of surgery (min)	282 [240; 322] (*N* = 40)	270 [240; 300] (*N* = 45)	274 [240; 312] (*N* = 48)
Intraoperative bleeding (ml)	400 [288; 800] (*N* = 40)	400 [250; 700] (*N* = 45)	600 [338; 948] (*N* = 48)
Crystalloids given (ml)	1600 [1200; 2100] (*N* = 40)	1500 [1150; 2100] (*N* = 45)	1500 [1200; 2225] (*N* = 48)
Blood transfusion (Yes)	0/40	0/45	0/48
Intraoperative total dose of fentanyl (μg)	500 [400; 562] (*N* = 40)	500 [500; 600] (*N* = 45)	500 [500; 605] (*N* = 48)

*Note*: Data availability is indicated in the corresponding cells. Abbreviations: ASA, American Society of Anesthesiologists; BMI, body mass index; Hb, haemoglobin; Hct, haematocrit; SA, systemic analgesia using lidocaine; SSS, spinal single shot; TAP, transversus abdominis plane block; Tc, thrombocytes.

### 4.1 QoR‐15 scores

**FIGURE 2:**
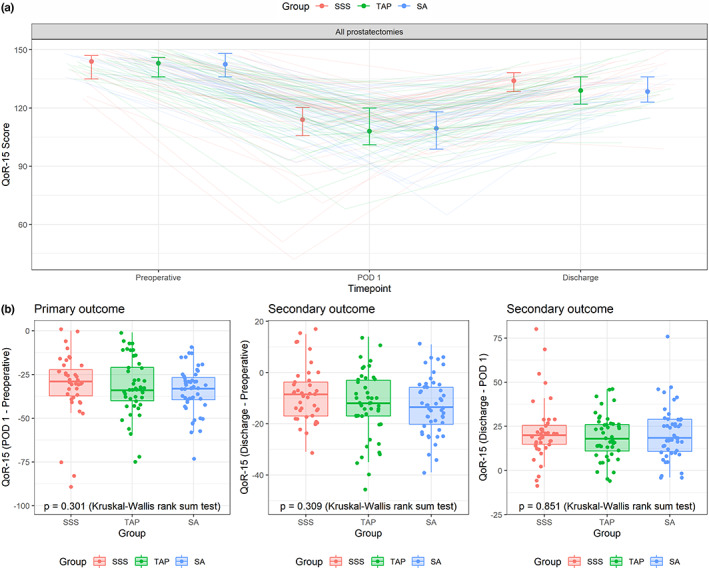
Quality of recovery (QoR‐15) scores for the entire cohort (including patients undergoing open and robotic‐assisted radical prostatectomy). (a) Time series of QoR‐15 scores for time points preoperative, postoperative and discharge. Median and interquartile ranges are shown as well as individual patients as coloured lines. (b) Primary outcome: Change in QoR‐15 postoperative minus preoperative. Secondary outcome: Change in QoR‐15 at discharge minus preoperative. Exploratory outcome: Change in QoR‐15 discharge minus postoperative. Box and whisker plots are shown as well as individual patients as coloured dots. Global *p*‐values from a Kruskal‐Wallis rank sum test are shown. No post‐hoc between‐group comparisons were computed as the global *p*‐values were not statistically significant.

**FIGURE 3:**
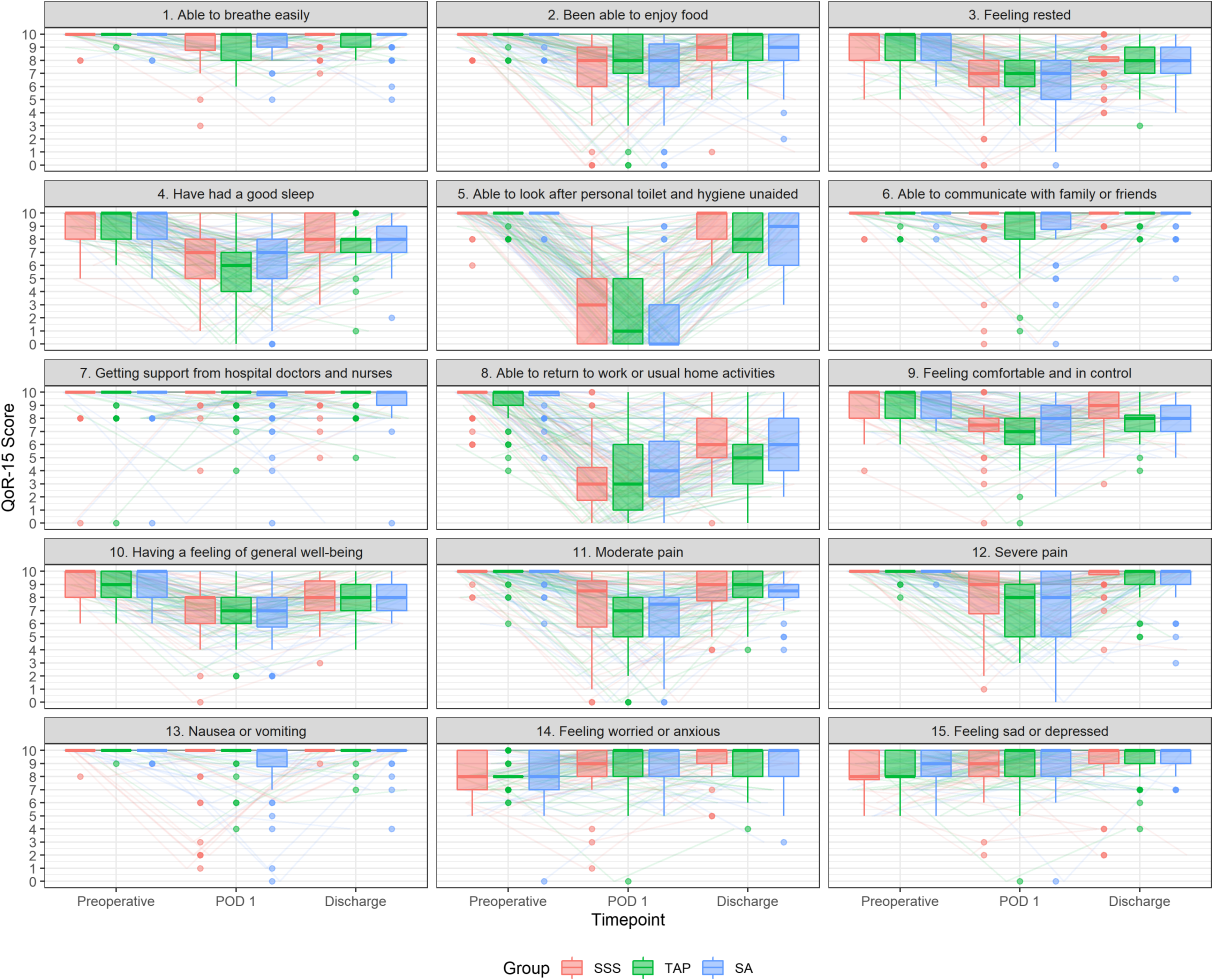
Box and whisker plot of the individual scores of the Quality of recovery (QoR‐15) questionnaire. Scores are shown for each treatment group (SSS, TAP and SA) for time points preoperative, postoperative and discharge. Coloured lines denote individual patients.

The pattern of QoR‐15 changes was similar in all three groups (Figure 2), even when analysing each question separately (Figure 3). QoR‐15 differences did not differ between the groups on POD 1 (Figure 2b, left diagram, global *p* = 0.301; effect size 0.003) or at hospital discharge (Figure 2b, middle diagram, global *p* = 0.309, effect size 0.003). No post‐hoc between‐group comparisons were computed as the global p‐values were not statistically significant. The median QoR‐15 score decrease on POD 1 was −29 (−37 to −22) in the SSS group, −34 (−40 to −21) in the TAP group and − 33 (−40 to −27) in the SA group (Table 2B). The median and IQR as well as the p‐value of a likelihood ratio test regarding the time and treatment group interaction in a generalized linear mixed model with a beta distribution of the QoR‐15 scores were not significant (*p* = 0.647). At discharge, the median QoR‐15 scores were good in all groups: SSS 134 (IQR 128–138); TAP 129 (IQR 122–136) and SA 128 (IQR 123–136) (Table 2A).

**TABLE 2:** QoR‐15 outcomes

(A) Summary statistics				
Treatment	Time points			
	Preoperative	POD 1	Discharge	*p*
SSS	144 [135; 147]	114 [106; 120]	134 [128; 138]	0.647
TAP	143 [136; 146]	108 [101; 120]	129 [122; 136]	
SA	142 [136; 148]	110 [98.8; 118]	128 [123; 136]	

(B) Outcomes and contrasts			
Treatment	Time points		
	POD 1—Preoperative	Discharge—Pre‐operative	Discharge—POD 1
Outcomes: QoR‐15 scores			
SSS	−29.0 [−37.25; −22.25]	−8.5 [−17.00; −3.75]	20.0 [14.8; 25.5]
TAP	−34.0 [−40.0; −21.0]	−12.0 [−17.0; −3.0]	18.0 [11.0; 26.0]
SA	−33.0 [−39.50; −26.75]	−13.5 [−20.25; −5.75]	18.5 [10.8; 29.0]
Contrasts: Difference in median QoR‐15 (95% CI)			
SA vs. SSS	−4.0 (−10.0; 1.0)	−4.0 (−8.0; 1.0)	−0.0 (−5.0; 5.0)
SA vs. TAP	−1.0 (−8.0; 4.0)	−1.0 (−6.0; 4.0)	1.0 (−4.0; 6.0)
SSS vs. TAP	3.0 (−4.0; 9.0)	3.0 (−2.0; 9.0)	1.0 (−4.0; 7.0)
Contrasts: *p*‐values (effect sizes)			
Global	0.301 (0.003)	0.309 (0.003)	0.851 (−0.013)

*Note*: (A) Descriptive statistics with median and interquartile range as well as the *p*‐value of a likelihood ratio test regarding the time and treatment group interaction in a generalized linear mixed model with transformed outcomes and a beta distribution (see methods). (B) Outcomes with associated *p*‐values from a global test (Kruskal‐Wallis Rank Sum Test). Effect sizes are given as η^2^ based on the H‐statistic for the global test.

Abbreviations: POD, postoperative day; SA, systemic analgesia using lidocaine; SSS, spinal single shot; TAP, transversus abdominis plane block.

When comparing subgroups undergoing open or robotic‐assisted prostatectomy, the postoperative decrease in QoR‐15 scores seemed more pronounced after ORP (Figure S1) than after RARP (Figure S2), albeit without statistical significance. A descriptive presentation of all 15 questions for the complete cohort is presented in Figure 3, stratified to ORP in Figure S3 and RARP in Figure S4.

### 4.2 Secondary outcomes

Pain scores were low in all groups and showed a similar pattern over time (Table 3). The median NRS score at rest 6 h postoperatively was 2 in all groups and their time evolution did not differ across treatment groups (*p* = 0.210). Pain during coughing or mobilization at rest 6 h postoperatively was 4 in all groups and did not differ across treatment groups (*p* = 0.601). Although the number of patients requiring rescue analgesics on POD 1 did not differ across treatment groups (*p* = 0.228), it differed on POD 2 (*p* = 0.041).

**TABLE 3:** Secondary outcomes (pain, rescue analgesics, fentanyl)

	6 h	POD 1	POD 2	*p*
Pain at rest (NRS)				
SSS	2.00 [0.00; 2.00]	1.00 [0.00; 2.25]	1.00 [0.00; 2.00]	0.210
TAP	2.00 [0.00; 2.50]	2.00 [1.00; 4.00]	2.00 [0.00; 3.00]	
SA	2.00 [1.00; 2.00]	2.00 [1.00; 3.25]	2.00 [0.00; 2.00]	
Deep visceral pain (NRS)				
SSS	2.00 [2.00; 4.00]	2.00 [2.00; 4.00]	2.00 [2.00; 2.25]	0.492
TAP	4.00 [2.00; 4.00]	4.00 [2.00; 5.00]	3.00 [2.00; 4.00]	
SA	3.00 [2.00; 4.00]	3.50 [2.00; 5.00]	3.00 [2.00; 4.00]	
Pain during mobilization/coughing (NRS)				
SSS	4.00 [2.00; 5.50]	4.00 [2.00; 5.00]	2.00 [2.00; 4.00]	0.601
TAP	4.00 [4.00; 6.00]	5.00 [3.50; 7.00]	4.00 [2.00; 5.00]	
SA	4.00 [3.00; 4.00]	4.00 [3.00; 6.00]	4.00 [3.00; 6.00]	
Rescue analgesics (Yes)				
SSS	27/39 (69.2%)	20/30 (50.0%)	9/40 (22.5%)	0.096
TAP	33/45 (73.3%)	30/45 (66.7%)	19/45 (42.2%)	
SA	28/47 (59.6%)	25/48 (52.1%)	23/48 (47.9%)	
Fentanyl (Yes)				
SSS	27/38 (71.1%)	13/40 (32.5%)	0/40 (0.00%)	0.021^a^
TAP	35/45 (77.8%)	23/44 (52.3%)	0/44 (0.00%)	
SA	28/47 (59.6%)	10/47 (21.3%)	3/47 (6.38%)	

*Note*: The *p*‐value of a likelihood ratio test regarding the time and treatment group interaction is derived with a generalized linear mixed model with transformed outcomes and a beta distribution (see methods).

Abbreviations: POD, postoperative day; SA, systemic analgesia using lidocaine; SSS, spinal single shot; TAP, transversus abdominis plane block.

^a^ Numerical instability due to complete separation in POD 2. Sensitivity analysis without POD2 resulted in a *p* = 0.587.

The number of patients requiring additional doses of fentanyl 6 h postoperatively was *27* (71.1%) in the SSS group, *35* (77.8%) in the TAP group and *28* (59.6%) in the SA group with no differences between the treatment groups (*p* = 0.153). On POD 2, *3* (6.4%) patients in the SA group required 25 μg i.v. fentanyl before being transferred to the ward.

Episodes of PONV within 6 h postoperatively were similar among the groups: SSS *2* (5.1%), TAP *2* (4.65%) and SA *7* (14.9%) (*p* = 0.153). Return of bowel function, including onset of first flatus, defecation and tolerance to food, was within 3 days in all groups and did not differ between the treatment groups (*p* = 0.085) (Table 4).

**TABLE 4:** Secondary outcomes (PONV, gastrointestinal function)

	SSS	TAP	SA	*p*
PONV (Yes)				
6 h	2/39 (5.13%)	2/43 (4.65%)	7/47 (14.9%)	0.807
POD 1	7/38 (18.4%)	8/45 (17.8%)	10/46 (21.7%)	
POD 2	1/35 (2.86%)	1/43 (2.33%)	4/46 (8.70%)	
POD 3	1/39 (2.56%)	1/42 (2.38%)	3/46 (6.52%)	
Gastrointestinal function				
Returned (Yes)	40/40 (100%)	45/45 (100%)	47/48 (97.9%)	>0.99
First day of return				0.085
POD 1	31/40 (77.5%)	31/45 (68.9%)	41/47 (87.2%)	
POD 2	8/40 (20.0%)	14/45 (31.1%)	6/47 (12.8%)	
POD 3	1/40 (2.50%)	0/45 (0.00%)	0 (0.00%)	
Flatus				
Occurred (Yes)	40/40 (100%)	45/45 (100%)	48/48 (100%)	1.0
First day of flatus				0.175
POD 1	30/40 (75.0%)	30/45 (66.7%)	37/48 (77.1%)	
POD 2	9/40 (22.5%)	15/45 (33.3%)	8/48 (16.7%)	
POD 3	1/40 (2.50%)	0/45 (0.00%)	3/48 (6.25%)	
Defecation				
Occurred (Yes)	38/40 (95.0%)	40/45 (88.9%)	47/48 (97.9%)	0.189
First day of defecation				0.292
POD 1	5/40 (13.2%)	10/45 (25.0%)	12/47 (25.5%)	
POD 2	21/40 (55.3%)	23/45 (57.5%)	20/47 (42.6%)	
POD 3	12/40 (31.6%)	7/45 (17.5%)	15/47 (31.9%)	
Tolerance to solid food				
Occurred (Yes)	40/40 (100%)	45/45 (100%)	48/48 (100%)	1.0
First day of tolerance				0.037
POD 1	38/40 (95.0%)	37/45 (82.2%)	46/48 (95.8%)	
POD 2	2/40 (5.00%)	8/45 (17.8%)	2/48 (4.17%)	
POD 3	0/40 (0%)	0/45 (0%)	0/48 (0%)	

*Note*: The *p*‐value of a likelihood ratio test regarding the time and treatment group interaction is derived with a generalized linear mixed model with transformed outcomes and a beta distribution (see methods section).

Abbreviations: POD, postoperative day; PONV, postoperative nausea and vomiting; SA, systemic analgesia using lidocaine; SSS, spinal single shot; TAP, transversus abdominis plane block.

## 5 DISCUSSION

### 5.1 Key findings

The decrease in QoR‐15 scores on POD 1 (primary outcome) did not differ between the three analgesic procedures (intrathecal administration of local anaesthetic and opioid, TAP block or systemic lidocaine administration) in this randomized controlled clinical trial. QoR‐15 differences between treatment groups did not reach the predefined minimal clinically important difference of 10 at any time point, even not when applying the recently proposed value of 6 (Myles, 2021).

We observed a similar longitudinal pattern of QoR‐15 decrease on POD 1 and recovery until discharge with no significant differences between groups. A median QoR‐15 score of above 122 at discharge illustrates a good recovery in all groups (Myles et al., 2016). On POD 1, questions 5 (personal toilet and hygiene) and 8 (return to work or usual home activities) had the biggest impact on QoR‐15 scores. At discharge, this was only due to question 8. The same pattern was present in both surgical subgroups (ORP and RARP).

Secondary outcomes, such as pain, PONV and return of bowel function did not differ between groups. Overall, pain scores were low at rest and during coughing or mobilization in all groups.

### 5.2 Relationship with previous studies

Numerous RCTs showed that multimodal concepts reduce the need for systemic analgesia after ORP or RARP (Andrieu et al., 2009; Brown et al., 2004; Ripolles et al., 2015; Shim et al., 2020; Wu et al., 2005). However, the literature is scarce investigating their impact on quality of recovery as measured by the QoR‐15 questionnaire. Our observations are comparable to those of Koning et al. (Koning et al., 2020). They showed that QoR‐15 was decreased by 10% after RARP in patients who received intrathecal bupivacaine and hydromorphone and by 13% in patients without intrathecal analgesia. At discharge, the QoR‐15 scores did not differ significantly between the groups, confirming our findings of comparable recovery.

The QoR‐15 questionnaire is a validated questionnaire commonly used in the perioperative setting and recommended as an outcome measure by the ESA‐ESICM joint task force on perioperative outcome measures (Jammer et al., 2015). The additional assessment of ‘traditional’ outcomes (opioid consumption, NRS scores, PONV, return of bowel function) in addition to the QoR‐15 was thought to reduce the risk of false‐negative interventions and bias.

One can argue that for the control group, represented by our standard‐of‐care approach, either baseline analgesia or the use of the systemic administration of lidocaine already leads to good results in terms of quality of recovery and pain control. This could render the statistical relevance and clinical justification of a more invasive approach like intrathecal analgesia or TAP block more challenging. Lidocaine is an amide local anaesthetic with analgesic, antihyperalgesic and anti‐inflammatory properties. Systemic administration of lidocaine has been found to reduce pain scores and be opioid‐sparing in abdominal and urological surgery in the last decades (Daykin, 2017; Groudine et al., 1998; Kaba et al., 2007). Arguably, in the SA group, lidocaine was administered over 24 h compared to single‐shot administration in the SSS and TAP groups, potentially confounding the comparison. Nevertheless, TAP block could serve as a valuable alternative in case of contraindication for intravenous lidocaine administration such as allergies or epileptic disposition. It is less invasive than spinal anaesthesia and more comfortable for the patient due to its application after induction of general anaesthesia.

We found similar intra‐operative fentanyl administration rates in all three groups. The additional use of intrathecal analgesia or a TAP block did not reduce the amount of fentanyl administered. This could be explained either by a good analgesic effect of systemic continuous administration of lidocaine or by pain originating from areas not covered by the loco‐regional techniques used. In addition, the long duration of surgery might have resulted in a reduced or insufficient regional block or intrathecal co‐analgesic effect towards the end of surgery.

This study showed similar pain scores after ORP and RARP, as no differences in NRS or the use of rescue analgesia were observed. We found pain intensity scores of approximately 4 to 5 on the numeric rating scale during coughing or mobilization, which is comparable to a previous multicentre cohort series (Gerbershagen et al., 2013). The incidence of PONV and return of bowel function were also similar. Return of bowel function within 3 days in all patients illustrates the low risk of delayed return of gastrointestinal function or ileus after ORP or RARP when using a multimodal approach and probably the low risk for ileus and delayed return of gastrointestinal function in this surgery. This can be explained with the strict extra‐peritoneal approach in ORP patients on one side and with the minimal trans‐peritoneal approach in RARP patients, lowering the damage of the peritoneum, a known risk factor for delayed return of bowel function. This could also explain the low incidence of PONV in all groups. In addition, intrathecal analgesia, TAP block and systemic administration of lidocaine resulted in similar QoR‐15 changes after ORP and RARP. It is worth mentioning the very similar course of QoR‐15 scores from POD 1 to recovery until discharge after ORP and RARP. It seems that the invasiveness of the surgical approach has only a limited influence on early recovery, if measured using a multidimensional score. Overall, the postoperative need for rescue analgesia was low within 6 h after surgery. Our results are in line with those of others originating from a high caseload prostate cancer centre, showing that pain levels are similar after ORP and RARP (Knipper et al., 2020).

### 5.3 Limitations

First, the lack of double blinding could be considered a limitation, as patients and anaesthesiologists in charge were not blinded to the allocated intervention. However, it is the state of the art to administer analgesics intrathecally in awake and cooperative patients. As the effect of intrathecal local anaesthetics is almost immediate, the patient's feedback will be prompt. In addition, the intrathecal injection of placebo is ethically questionable.

Second, a relevant number of patients refused the allocated intervention (SSS), despite having provided consent beforehand. This reflects the low acceptance, mostly due to a fear, of intrathecally administered concomitant analgesia, thus limiting the implementation of this technique in clinical practice.

In addition, data from patients either refusing spinal anaesthesia or presenting with anatomy making TAP block application impossible were not collected. Therefore, performing an intention‐to‐treat analysis was not possible.

Third, the clinical effect of the TAP block could not be assessed due to application after induction of anaesthesia. This limitation has been addressed by sonographic confirming of spread of local anaesthetic in the correct layer.

Fourth, one intervention (SA) was given over 24 h, whereas the other two (TAP, SSS) were applied at a single time point. In addition, the effect of the TAP block is expected to last longer due to the clonidine applied in comparison with a spinal anaesthesia using fentanyl and not longer‐lasting morphine.

Fifth, due to the design of the study, it cannot be differentiated, which part of the multimodal analgesia regimen used (baseline analgesia or the applied additional technique) is mainly responsible for good quality of recovery.

Sixth, data from this centre regarding the size and distribution of QoR‐scores were unknown at the time of study planning, so sample size calculation had to be based on previously published studies (Myles, 2016a).

### 5.4 Conclusion

The comparison between a single shot of intrathecal bupivacaine and fentanyl, a TAP block with ropivacaine and clonidine and systemic administration of lidocaine over 24 h showed no significant difference in quality of recovery on POD 1 assessed by the QoR‐15 questionnaire, postoperative pain scores, intra‐ and postoperative opioid administration, PONV or return of bowel function. Quality of recovery at discharge was considered as good in all three groups.

## AUTHOR CONTRIBUTIONS

D. Engel and P. Wuethrich came up with the study idea. All authors contributed to the study design, data collection and interpretation. All authors discussed the results, commented on the manuscript and gave final approval.

## FUNDING INFORMATION

This study was supported by internal institutional research funds from the Department of Anaesthesiology and Pain Medicine, University Hospital Bern, Bern, Switzerland; and from an independent foundation, the ‘Stiftung für die Forschung in Anästhesiologie und Intensivmedizin’ (‘Foundation for Research in Anaesthesiology and Intensive Care’, 3010 Bern [grant number 31/2019]). None of the funding institutions had any role in the design and conduct of the study; collection, management, analysis or interpretation of the data; preparation, review, or approval of the manuscript; or decision to submit the manuscript for publication.

## TRIAL REGISTRATION

Clinicaltrials.gov identifier: NCT03618693.

## Supporting information


Figure S1

Figure S2

Figure S3

Figure S4
