# Supplementary figures and images for: Impact of analgesic techniques on early quality of recovery after prostatectomy: A 3‐arm, randomized trial
Source: Eur J Pain. 2022 Aug 21;26(9):1990–2002. doi: 10.1002/ejp.2020 (PMC9541353; doi:10.1002/ejp.2020)

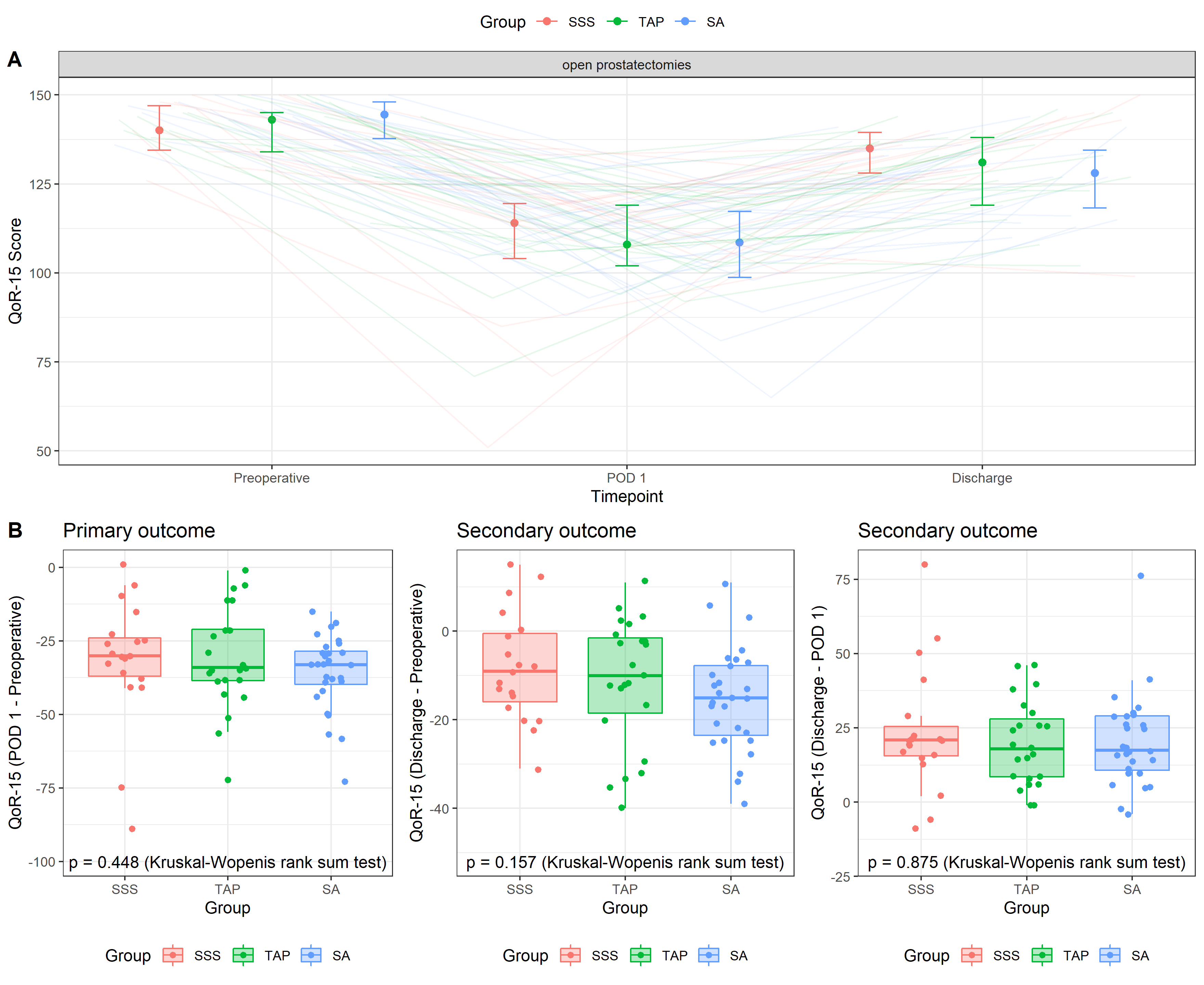

Supplement: Supplementary file 1 — Figure S1 [file EJP-26-1990-s002.png]

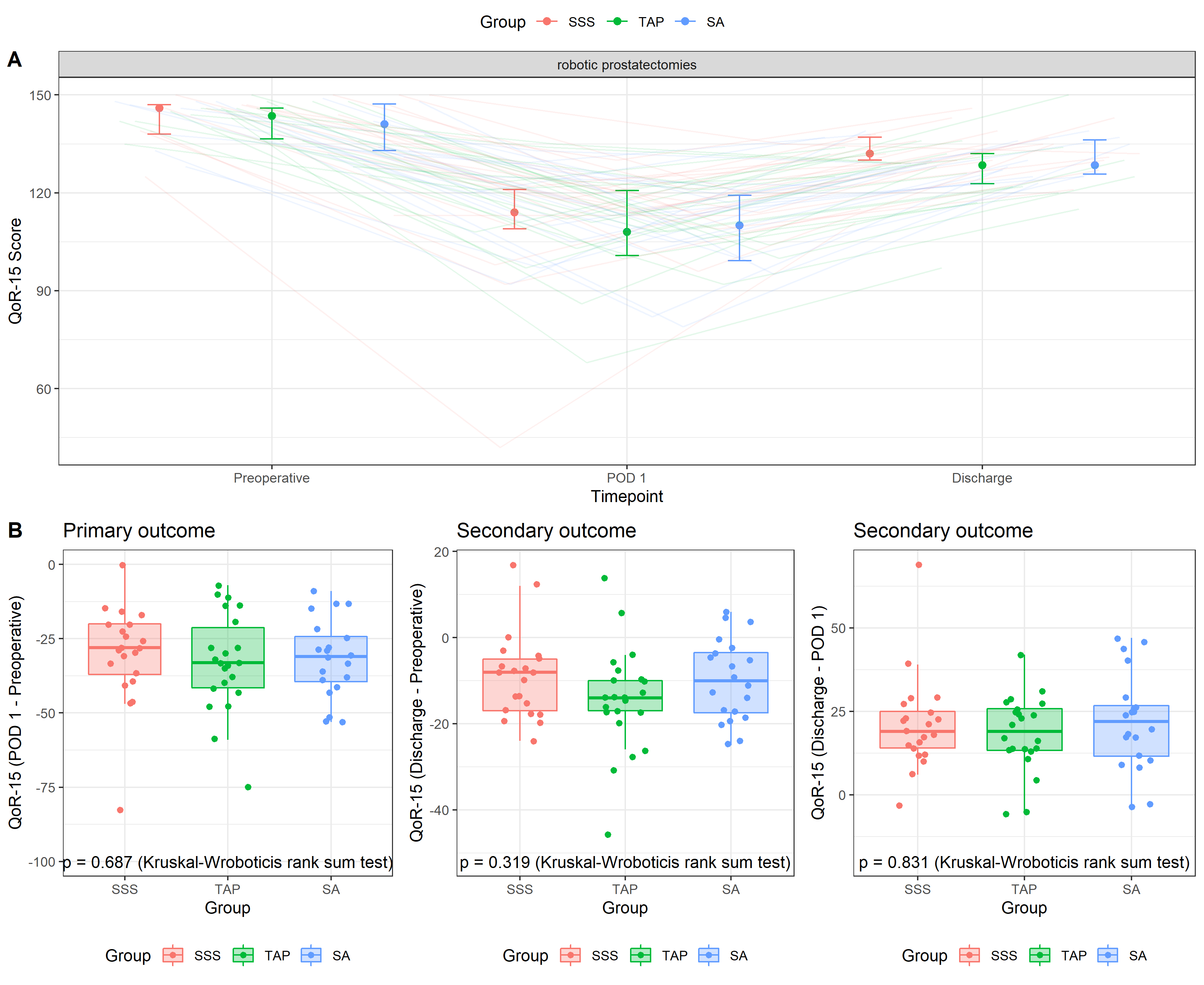

Supplement: Supplementary file 2 — Figure S2 [file EJP-26-1990-s003.png]

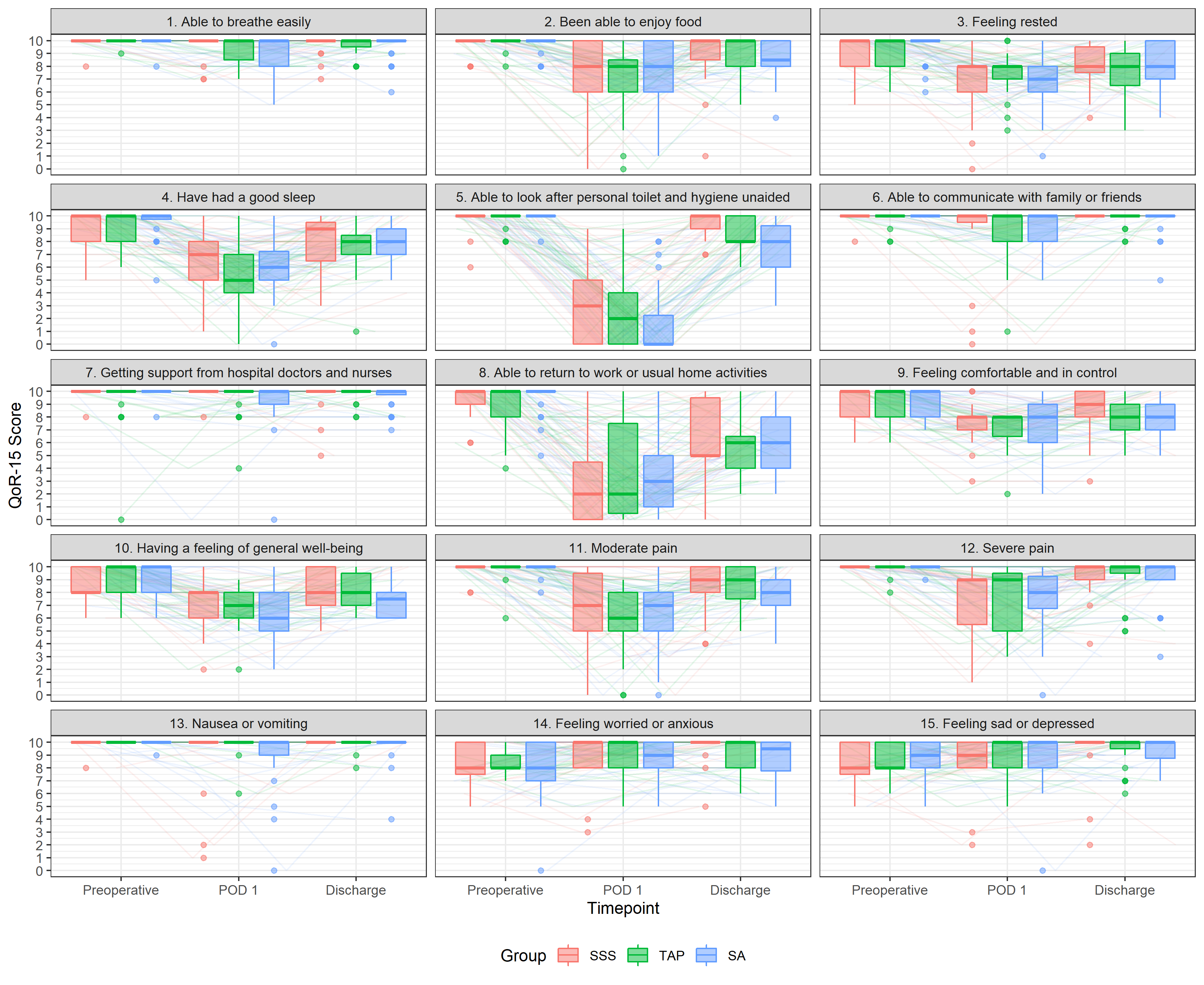

Supplement: Supplementary file 3 — Figure S3 [file EJP-26-1990-s004.png]

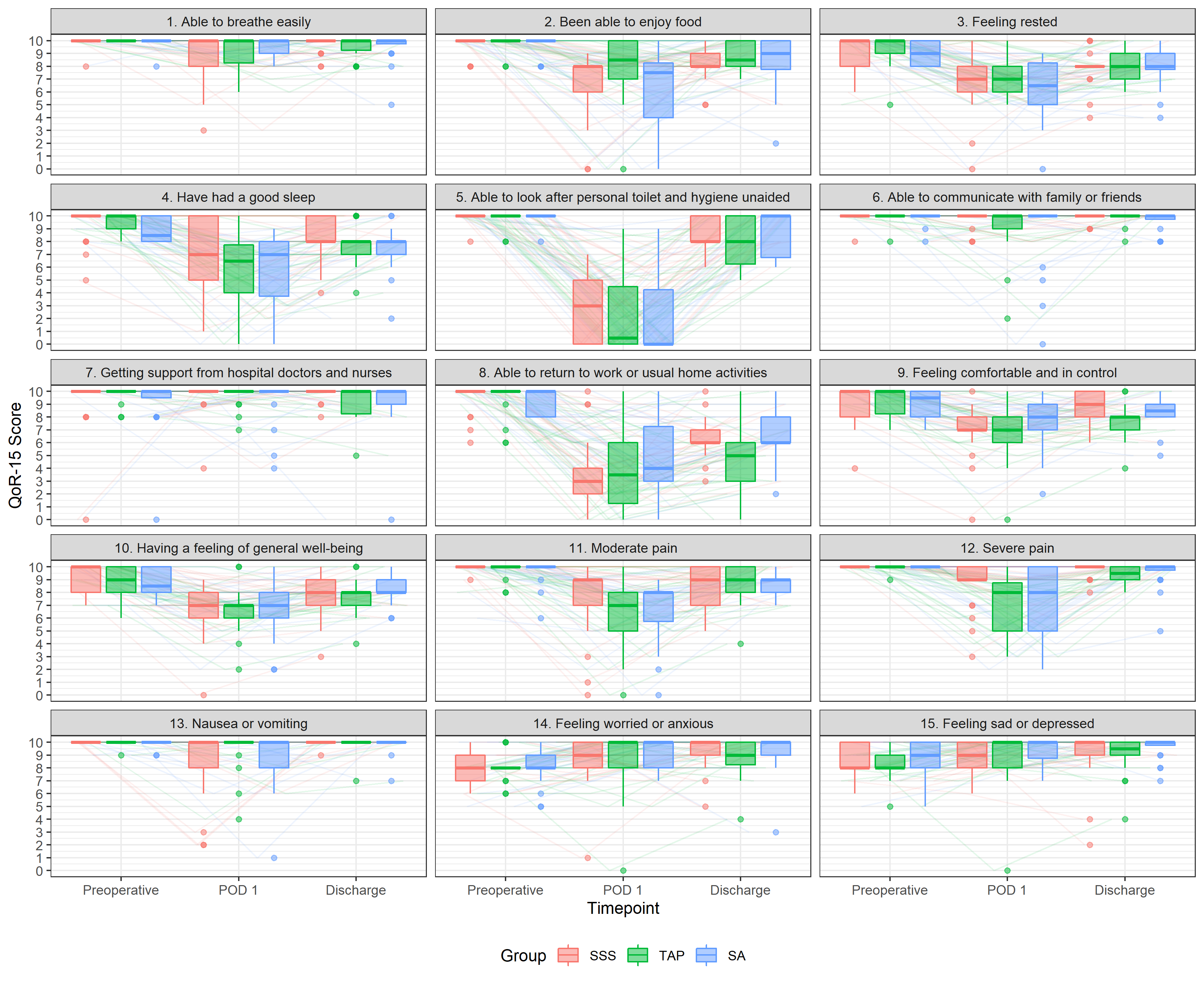

Supplement: Supplementary file 4 — Figure S4 [file EJP-26-1990-s001.png]
